# Advanced Biliary Cannulation Masterclasses Improve Self-reported Knowledge and Teaching Confidence in Needle-knife Fistulotomy

**DOI:** 10.1055/a-2965-6853

**Published:** 2026-09-30

**Authors:** Shabbir Jivanjee, Damien Durkin, Noor H. Bekkali, Sajjad Mahmood, Umesh Basavaraju, Vikramjit Mitra, Oliver Reed, John Anderson, Trevor Brooklyn, Srisha Hebbar

**Affiliations:** 1 Department of Gastroenterology and Endoscopy 9898 University Hospitals of North Midlands NHS TrustStoke-on-TrentEnglandUnited Kingdom of Great Britain and Northern Ireland; 2 Department of Gastroenterology 11269 John Radcliffe HospitalOxfordOxfordshireUnited Kingdom of Great Britain and Northern Ireland; 3 Department of Gastroenterology 159812 Wythenshawe HospitalManchesterEnglandUnited Kingdom of Great Britain and Northern Ireland; 4 Department of Gastroenterology 1015 NHS GrampianAberdeenScotlandUnited Kingdom of Great Britain and Northern Ireland; 5 Department of Gastroenterology 3762 North Tees and Hartlepool NHS Foundation TrustHartlepoolEnglandUnited Kingdom of Great Britain and Northern Ireland; 6 Department of Gastroenterology 1602 Belfast City Hospital Health and Social Services TrustBelfastNorthern IrelandUnited Kingdom of Great Britain and Northern Ireland; 7 Department of Gastroenterology 156720 Cheltenham General HospitalCheltenhamGloucestershireUnited Kingdom of Great Britain and Northern Ireland; 8 Department of Gastroenterology 156720 Cheltenham General HospitalCheltenhamEnglandUnited Kingdom of Great Britain and Northern Ireland; 9 Department of Gastroenterology 9898 University Hospitals of North Midlands NHS TrustStoke-on-TrentUnited Kingdom of Great Britain and Northern Ireland

**Keywords:** pancreatobiliary (ERCP/PTCD), ERC topics, stones, quality and logistical aspects, training

## Abstract

**Background and Aims** Needle-knife fistulotomy (NKF) is widely used for difficult biliary access, yet formal training is inconsistent and teaching confidence remains low. We aimed to characterise baseline NKF experience among independent endoscopic retrograde cholangiopancreatography (ERCP) practitioners and evaluate the self-reported educational impact of a national advanced biliary cannulation masterclass series.

**Materials and Methods** Ten 1-day masterclasses were delivered for 152 independent ERCP practitioners, combining video-based case discussion, didactic teaching, and supervised hands-on simulator training. Pre-course questionnaires captured baseline NKF use. Post-course questionnaires used a retrospective before-and-after format across six cannulation domains. Wilcoxon signed-rank tests were applied.

**Results** Of 126 pre-course respondents, 82.5% had used NKF, but only 33.3% had received formal training and 27.0% had trained a trainee. Prospective pre-course NKF knowledge was 5.8/10 and teaching confidence was 3.7/10. On retrospective post-course assessment ( *n* = 122), median NKF knowledge increased from 5/10 (interquartile range [IQR], 4–7) to 9/10 (IQR, 8–9; *p* < 0.001) and teaching confidence increased from 4/10 to 7/10 ( *p* < 0.001); the proportion rating ≥7/10 rose from 14% to 53%. Post-course, 94.3% intended to use NKF more often, and 79.8% would consider primary fistulotomy in selected cases.

**Conclusions** NKF is commonly used despite limited formal training and low teaching confidence. A national masterclass series was associated with improvements in self-reported knowledge and teaching confidence. A planned longitudinal prospective audit will determine whether these gains translate into changes in practice and patient outcomes.

## Introduction

Selective deep biliary cannulation fails in 5–15% of cases even in high-volume endoscopic retrograde cholangiopancreatography (ERCP) centres, and post-ERCP pancreatitis (PEP) remains a common serious adverse event. 1 Repeated ampullary trauma during prolonged cannulation attempts is an independent risk factor for PEP, supporting timely escalation when standard access proves difficult. 2 The British Society of Gastroenterology ERCP Quality Improvement Programme recommends protected ERCP-specific continuing professional development (CPD), including a minimum of four hours per month. 3

The European Society of Gastrointestinal Endoscopy (ESGE) guidelines define difficult biliary cannulation using the “5-5-1” rule: more than five papillary contacts, more than 5 minutes of cannulation time, or more than one unintended pancreatic duct cannulation. 1 While the ESGE algorithm recommends specific rescue approaches in selected scenarios, technique selection across a broader range of ampullary morphologies remains operator dependent. A recent network meta-analysis of 12 randomised controlled trials suggested that needle-knife approaches may offer advantages over prolonged standard cannulation attempts, although no single rescue strategy was universally superior. 4

Needle-knife fistulotomy (NKF), in which suprapapillary access is created above the native papillary orifice, has been evaluated both as an early rescue technique and, in selected cases, as a primary access strategy. Meta-analyses suggest that early precut access does not increase procedural risk compared with prolonged standard cannulation attempts, 5 and primary fistulotomy has demonstrated high technical success with low pooled PEP rates in prospective studies. 6 Ampullary morphology may also influence cannulation difficulty and adverse event risk, and the Haraldsson classification provides a reproducible framework for describing major ampullary morphology. 7 8

Despite this evidence base, formal training in NKF remains inconsistent. Advanced rescue cannulation techniques are often acquired informally after completion of standard ERCP training, and no structured national training programme for NKF has previously been described. We therefore designed and delivered a national advanced biliary cannulation masterclass series and evaluated baseline training experience and post-course educational outcomes among independent ERCP practitioners.

## Methods

### Course Design and Delivery

**Video 1** Demonstration of needle-knife fistulotomy training using the modified Boškoski–Costamagna ERCP Trainer and a synthetic ERCP simulator platform during the advanced biliary cannulation masterclass series. The video illustrates faculty-guided instruction in scope positioning, needle alignment, suprapapillary incision orientation, and morphology-informed technique discussion.


Ten 1-day advanced biliary cannulation masterclasses were delivered between December 2024 and November 2025 across regional endoscopy centres in Stafford, Oxford, Stockton-on-Tees, Dunblane, Manchester, Belfast, Bath, and Torquay. The target audience was independent ERCP practitioners. All delegates were unique individuals and no participant attended more than one session.

Each masterclass accommodated 10–18 delegates with an approximate faculty-to-delegate ratio of 1:3–4. Curriculum consistency was maintained through a standardised slide set and annotated video library used at all sites.

The curriculum covered the ESGE 5-5-1 escalation framework, double guidewire technique, transpancreatic sphincterotomy, periampullary diverticular cannulation, ampullary morphology using the Haraldsson classification, and morphology-guided NKF strategy. Educational delivery combined peer discussion, didactic teaching, annotated video review, and supervised simulator-based practice.

Hands-on training used two complementary ERCP simulator platforms: a modified Boškoski–Costamagna ERCP Trainer and the Mikoto synthetic ERCP model. These platforms were used to demonstrate scope positioning, needle alignment, controlled suprapapillary incision, and faculty-supervised deconstruction of NKF technique ( **Video 1** ). No formal summative competency assessment, structured knowledge test, DOPS, or TEESAT-based assessment was performed.

### Data Collection

Prior to each session, delegates completed a prospective pre-course questionnaire assessing prior NKF use, formal NKF training, primary fistulotomy experience, previous trainee teaching experience, and self-rated confidence in teaching trainees using a 1–10 Likert scale. Self-rated NKF knowledge was measured using the same scale. Equivalent paper-based questionnaires were used at earlier sessions where online collection systems were unavailable.

The questionnaires were developed by the faculty for service-evaluation purposes and were not previously validated.

Immediately after course completion, delegates completed a post-course questionnaire using retrospective before-and-after self-reported scores across six domains: NKF knowledge, ampullary morphology, confidence in training trainees, diverticular cannulation, transpancreatic sphincterotomy, and double guidewire technique. This retrospective pre/post design was chosen to reduce baseline overestimation; however, it remains susceptible to recall bias, response-shift bias, social desirability bias, and inflation of perceived effect sizes. Additional items captured intended practice change and morphology-related indications for future NKF use.

For the primary paired analysis, retrospective pre-course scores from the post-course questionnaire were matched with corresponding post-course scores. Complete paired data were available for 124 respondents; two were excluded after data-quality review because of implausible uniform responses across all domains, yielding *n* = 122 for the primary paired analysis. The two excluded respondents provided valid responses to intended practice-change items and were therefore retained within denominators for those analyses ( *n* = 124). Double guidewire technique and transpancreatic sphincterotomy items were introduced after the first two sessions ( *n* = 81 pairs).

### Statistical Analysis

Descriptive statistics are reported for baseline and outcome variables. Paired ordinal pre- and post-course Likert scores were analysed using the Wilcoxon signed-rank test. Medians and interquartile ranges (IQRs) were used as primary summary measures.

Effect size was expressed using the standardised response mean (SRM = mean difference / standard deviation of differences), classified as moderate (≥0.5), large (≥0.8), or very large (>1.0). Bonferroni correction was applied for six parallel comparisons (adjusted significance threshold α = 0.0083).

Analyses were performed using Python version 3.12. Analyses used complete-case paired data only.

This study was conducted as a service evaluation according to UK Health Research Authority guidance and did not require formal Research Ethics Committee review.

## Results

### Participants

A total of 152 independent ERCP practitioners attended the ten national training sessions. Pre-course questionnaires were completed by 126 participants (82.9%) and post-course questionnaires were completed by 124 participants, of whom 122 were included in the primary paired analysis after exclusion of two implausible uniform-response datasets. Double guidewire technique and transpancreatic sphincterotomy analyses included 81 paired responses.

Baseline characteristics and pre-course experience are summarised in Table 1 .

**Table 1:** Participant characteristics and pre-course experience.

Characteristic	Value
**Programme delivery**	
Masterclasses delivered, *n*	10
Locations	Stafford (3), Oxford, Stockton-on-Tees, Dunblane, Manchester, Belfast, Bath, Torquay
Total participants enrolled	152
Delegates per session	10–18
All participants were unique individuals	Yes
Pre-course questionnaire responses	126 (82.9%)
Post-course questionnaire responses	124 (81.6%)
** Prospective pre-course self-assessment ( *n* = 126) **	
NKF knowledge, mean/10 (median; IQR)	5.8 (6; 5–8)
Confidence in training trainees, mean/10 (median; IQR)	3.7 (3; 2–5)
Confidence in training trainees ≥7/10	14% (18/126)
**Baseline clinical experience**	
Had used NKF for ductal access	82.5% (104/126)
Had performed primary fistulotomy	42.1% (53/126)
Had received formal NKF training	33.3% (42/126)
Had trained trainees in NKF	27.0% (34/126)

### Baseline NKF Experience and Training

On the prospective pre-course questionnaire, 82.5% (104/126) had used NKF and 42.1% (53/126) had performed primary fistulotomy. However, formal training was uncommon: only 33.3% (42/126) had received formal NKF training and 27.0% (34/126) had trained a trainee in the technique.

Mean NKF knowledge was 5.8/10 (median 6; IQR 5–8) and confidence in training trainees was 3.7/10 (median 3; IQR 2–5), with only 14% (18/126) rating confidence ≥7/10.

### Post-course Self-reported Scores

Self-reported scores improved across all six domains on retrospective post-course assessment ( Table 2 ; all *p* < 0.001; all remained significant following Bonferroni correction).

**Table 2:** Pre- and post-course self-reported scores by domain.

Technical domain	*n*	Pre-course median (IQR)	Post-course median (IQR)	Median change	SRM	*p* Value
Ampullary morphology	122	6 (5–7)	9 (8–10)	+3	1.82	<0.001
NKF knowledge	122	5 (4–7)	9 (8–9)	+3	1.78	<0.001
Diverticular cannulation	122	7 (5–8)	8 (8–9)	+2	1.19	<0.001
Confidence in training trainees	122	4 (2–6)	7 (4–8)	+2	1.08	<0.001
Transpancreatic sphincterotomy	81	4 (2–6)	7 (5–8)	+2	1.04	<0.001
Double guidewire technique	81	8 (7–9)	8 (8–9)	+0.5	0.75	<0.001

NKF knowledge ( *n* = 122 pairs) increased from a median of 5/10 (IQR, 4–7) to 9/10 (IQR, 8–9; median change +3; SRM 1.78). Ampullary morphology demonstrated a similarly large improvement (SRM 1.82; median change +3). Confidence in training trainees improved from 4/10 to 7/10 (SRM 1.08), and the proportion rating confidence ≥7/10 increased from 14% to 53%. Double guidewire technique demonstrated the smallest change, consistent with a ceiling effect.

### Intended Practice Change

Post-course, 94.3% of delegates stated that they intended to use NKF more often within their escalation pathway (25.4% significantly more often; 68.9% slightly more often) ( Table 3 ). The proportion willing to consider primary fistulotomy in selected cases was 79.8% (99/124).

**Table 3:** Intended practice change.

Post-course outcome	Response
Will use NKF significantly more often	25.4% (31/122)
Will use NKF slightly more often	68.9% (84/122)
Total: will use NKF more often	94.3% (115/122)
No intended change in NKF use	5.7% (7/122)
Will consider primary fistulotomy in selected cases ( *n* = 124)	79.8% (99/124)
NKF indication: bulging ampulla with failed standard cannulation ( *n* = 124)	33.1% (41/124)
NKF indication: combination of morphological types ( *n* = 124)	62.9% (78/124)

When asked about morphology-related indications, 62.9% (78/124) selected a combination of morphological types and 33.1% (41/124) selected bulging ampulla with failed standard cannulation alone.

### Delegate Feedback

Free-text comments were provided by 89 of 122 post-course respondents (73%). Delegates particularly valued annotated video-based case discussion, unedited adverse-event review, morphology-guided escalation frameworks, collegial peer-learning format, and supervised simulator practice. Requests for extended simulator-training time were common.

## Discussion

This national educational evaluation identified a substantial training gap in advanced biliary cannulation practice among independent ERCP practitioners in the United Kingdom. The principal finding was that NKF was already widely used in independent practice despite limited formal training exposure and low confidence in teaching the technique. These findings suggest that advanced rescue cannulation techniques are frequently acquired informally after completion of standard ERCP training.

The majority of delegates had already performed NKF prior to attending the programme, yet only one-third had received formal training and fewer than one-third had trained trainees in the technique. This discrepancy between widespread procedural adoption and limited structured pedagogy may have implications for procedural escalation thresholds, teaching consistency, and technical supervision.

Using Kirkpatrick’s educational evaluation framework, 9 this study primarily evaluates Level 2 educational outcomes, namely, perceived knowledge and self-reported confidence. These ratings do not establish objective knowledge, technical competence, or clinical performance. Intended practice changes should not be interpreted as evidence of actual behavioural modification or improved patient outcomes. Similarly, increased confidence in training trainees reflects self-perception rather than competence measured using structured knowledge testing, DOPS, 13 TEESAT, 14 simulator performance metrics, or observed clinical outcomes.

The curriculum emphasised morphology-informed escalation and the importance of balancing indications, contraindications, and operator expertise. Early or primary NKF was discussed only in selected anatomical scenarios, particularly a Haraldsson type 3 protruding or pendulous papilla with a prominent intraduodenal segment and a clearly identifiable suprapapillary roof. Type 4 creased or ridged papillae may also provide a visible suprapapillary access plane in selected cases. By contrast, type 2 small or flat papillae were presented as less favourable for primary NKF because of limited tissue and a less clearly defined incision plane. Morphology was therefore taught as one component of decision-making rather than as a prescriptive indication, and the final choice of technique remains dependent on ductal anatomy, pancreatic access, procedural context, and operator experience. 7 8

The simulator component was designed to support supervised deconstruction of NKF technique, including scope positioning, knife alignment, controlled incision orientation, and discussion of escalation strategy. Existing evidence supports the educational value of ERCP simulation. A multicentre randomised trial of the Boškoski–Costamagna ERCP Trainer found higher native biliary cannulation success, shorter cannulation times, and faster improvement in several ERCP performance domains among novices receiving early simulator training, although the primary outcome of global competence was not significantly different. 10 Biological and synthetic papillae incorporated into this platform have also demonstrated face and content validity for sphincterotomy and precut training. 11 12 These models can reproduce important component skills and provide deliberate practice without patient risk, but their capacity to determine independent NKF competence remains unproven. The most useful simulators should permit realistic scope positioning and stability, reproducible papillary morphology, tactile and visual feedback, use of standard accessories, controlled electrosurgical incision, objective capture of errors and performance metrics, repeated practice, and graded task difficulty. They should be integrated with expert feedback and validated assessments rather than used as substitutes for supervised clinical experience.

The morphology-based framework should similarly be interpreted as an educational approach rather than as evidence that morphology-guided NKF improves clinical outcomes. While delegates reported intended changes in escalation strategy, actual procedural behaviour, cannulation success, complication rates, and patient-level outcomes were not assessed. Prospective studies should combine validated knowledge testing and simulator-based assessment with longitudinal DOPS 13 or TEESAT 14 evaluation and clinical metrics, including cannulation success, time to biliary access, pancreatic duct instrumentation, PEP, bleeding, perforation, repeat ERCP, and the need for alternative biliary drainage.

Several limitations should be acknowledged. First, baseline scores in the paired analysis were reconstructed retrospectively after the intervention. This design may reduce pre-course overestimation but introduces recall and response-shift bias; consequently, the magnitude of the observed SRMs may overstate the true educational effect and should not be interpreted as an objective measure of learning. Second, all outcomes were self-reported and no validated knowledge test, DOPS, 13 TEESAT 14 assessment, blinded simulator rating, procedural metric, or patient outcome was collected. The study therefore demonstrates perceived educational benefit rather than improved technical competence or cannulation performance. Third, this was a single-arm educational evaluation of a relatively small, self-selected, technically engaged cohort without a comparator group. The changes cannot be attributed solely to the masterclass and may partly reflect participant engagement, peer interaction, expectations, or contextual effects. A controlled prospective design would strengthen causal inference. Fourth, immediate post-course assessment does not establish retention or transfer into clinical practice. Finally, longitudinal clinical follow-up data are not yet available. A planned 12-month prospective audit will assess NKF utilisation, timing of escalation, cannulation success, PEP, bleeding, perforation, repeat ERCP, and need for alternative biliary drainage.

## Conclusions

NKF is commonly used in independent ERCP practice despite limited formal training exposure and low confidence in teaching. A structured advanced biliary cannulation masterclass series was associated with improvements in perceived knowledge and self-reported teaching confidence. These findings support further evaluation of structured CPD programmes for advanced cannulation techniques but do not demonstrate improved technical competence or clinical outcomes. A planned longitudinal prospective audit will determine whether the reported gains translate into changes in practice and patient outcomes.

## Declaration of GenAI Use

During the preparation and revision of this manuscript, the authors used ChatGPT (OpenAI) and Claude (Anthropic) solely to improve language, readability, and clarity. All AI-assisted text was critically reviewed and edited by the authors, who take full responsibility for the accuracy, integrity, and final content of the manuscript.
